# New Route of Tire Rubber Devulcanization Using Silanes

**DOI:** 10.3390/polym15132848

**Published:** 2023-06-28

**Authors:** Rounak Ghosh, Christian Mani, Roland Krafczyk, Rupert Schnell, Alexander Paasche, Auke Talma, Anke Blume, Wilma K. Dierkes

**Affiliations:** 1 Department of Solids, Surfaces and Systems, Faculty of Engineering Technology, University of Twente, P.O. Box 217, 7500 AE Enschede, The Netherlands; a.g.talma-1@utwente.nl (A.T.); a.blume@utwente.nl (A.B.); 2 Evonik Industries, Paul-Baumann-Straße 1, 45772 Marl, Germany; christian.mani@evonik.com (C.M.); roland.krafczyk@evonik.com (R.K.); rupert.schnell@evonik.com (R.S.); alexander.paasche@evonik.com (A.P.)

**Keywords:** devulcanization, rubber recycling, silane, end-of-life tire, sustainability, circular economy

## Abstract

The disposal of tires at the end of their lifespan results in societal and environmental issues. To tackle this, recycling and reuse are effective solutions. Among various recycling methods, devulcanization is considered to be a very sustainable option, as it involves the controlled breakdown of crosslinks while maintaining the polymer backbones. The objective of this study is to develop a sustainable devulcanization process for passenger car tire rubber using silanes. In this study, a thermo-mechanical–chemical devulcanization process was conducted to screen six potential devulcanization aids (DAs). Silanes were chosen as they are widely used in tire rubber as coupling agents for silica. The efficiency of the devulcanization was studied by the degree of network breakdown, miscibility of the devulcanized material, and mechanical properties of the de- and revulcanized material. Compared to the parent compound, a 55–60% network breakdown was achieved for the devulcanizate along with 50–55% of tensile strength recovery. In addition to superior devulcanization efficiency, this DA offers a sustainable alternative to the conventional ones, such as di-phenyl-di-sulphide, due to its compliance with safety regulations. The devulcanizate can be utilized in high-performance applications, such as tires and seals, while 100% devulcanizate can be employed in low-strength technical rubber products.

## 1. Introduction

The extensive usage of rubber in various fields of application causes a serious environmental problem in terms of waste. In this scenario, the great challenge for civilization is to recycle and reuse this rubber [1]. Around 70% of the rubber production worldwide is consumed by the tire industry [2]. The use of styrene butadiene rubber (SBR) in tire production is increasing due the requirement of low rolling resistance, resulting in decreased fuel consumption and less carbon dioxide emissions [3]. Apart from tires, different molded and extruded rubber products, such as conveyor belts, hoses, etc., are also produced from SBR. The greatest hurdle for recycling is that rubber products are mostly vulcanized. During the vulcanization process, polymer chains are crosslinked, which provides a three-dimensional stable network. The insoluble and infusible nature of vulcanized rubber impedes the recycling process [1].

Landfilling of end-of-life tires (ELTs) is inhibited in the European Commission since 1999, which accelerated the recycling and reuse of ELTs in many European countries [4]. Most of the ELTs in Europe are granulated or ground to prepare floorings, walkway tiles, thermal isolation, acoustic isolation, road materials, and road as well as rail equipment [5]. Moreover, the usage of recycled rubber allows us to reduce the carbon footprint of rubber products [6].

**Figure 1:**
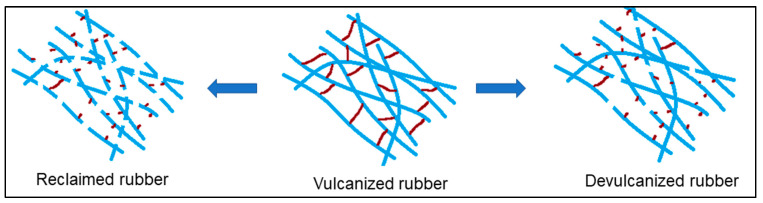
Schematic diagram of reclamation and devulcanization.

Different recycling methods for rubber are described in the literature [7,8], namely, mechanical [9,10,11,12], ultrasonic [13,14,15], chemical [16,17], microwave [18,19,20], cryogenic [21,22,23], microbial [24,25,26], thermo-mechanical–chemical [27,28,29], etc. Most of the recycling processes are mainly based on the random scission of the polymer network and crosslinks [4], whereas the selective breakdown of the crosslinks is required to improve the recycled rubber quality. The polymer chains have to stay intact as much as possible, which introduces the scope of devulcanization [3]. Devulcanized rubber depicts better mechanical properties than reclaimed rubber due to the higher percentage of intact polymer backbones [7]. Hence, devulcanization is considered the most sustainable solution for rubber recycling. A comparative schematic diagram of rubber reclamation and devulcanization is shown in Figure 1.

The most common and effective devulcanization mechanism is the rearrangement of the covalent bonds. Devulcanization aids (DAs) produce free radicals or reactive moieties during the devulcanization process, which react with the active groups of the broken crosslinks and form a new stable bond. Disulphides are the most common moieties for devulcanization, e.g., bis(3-triethoxysilyl propyl) disulphide (TESPD), diphenyl disulphide (DPDS), 2,2-dibenzamido-diphenyl disulphide (DBDPD), etc. [1]. A DA containing a polysulphide moiety is bis(3-triethoxysilyl propyl) tetrasulphide (TESPT) [2,6,27].

The filler system plays a vital role in the devulcanization process, as filler–polymer and filler–filler bonds become distorted during the thermo-mechanical process. For natural rubber (NR) filled with carbon black (CB), devulcanization is comparatively easier than for silica-filled rubbers: the breakdown of the physical interaction between the filler and polymer matrix is easier than the breakdown of the chemical bonds [1]. In tire technology, more and more tire treads contain a silica–silane filler system for reinforcement. Silanes are effective as bifunctional DAs for this type of rubber, as they potentially also reactivate the filler. For the silica-filled system, the breaking of the silica–polymer network is an additional challenge of the devulcanization process [28,29]: the controlled breakdown of polymer crosslinks and filler–polymer bonds keeping the polymer backbone intact. To date, there is a lack of a dedicated devulcanization process specifically designed for silica–silane-filled tires. The novelty of this study lies in addressing this gap by proposing an efficient devulcanization process design for silica-filled tires.

In this study, different silane-based DAs are screened and the devulcanizates are analyzed by the degree of network breakdown, mechanical properties of the revulcanized material, and miscibility of the devulcanized rubber with a virgin compound. The process can be described as the devulcanization of crosslinked polystyrene and polybutadiene rubbers reinforced by a silica–silane system. A silica-filled SBR-BR-based model tire tread compound was used as the feed material for the devulcanization process. Commercially available silane coupling agents with disulphide, polysulphide, amino, alkenyl, and mercapto moieties were selected as DAs for the screening trials. The DAs are selected considering the potentially active chemical moieties and processibility for devulcanization. Moreover, sustainability, environmental and health impacts, EU safety regulations, innovation, and commercial feasibility are considered for this development.

## 2. Materials and Methods

The experimental process can be classified into three parts:

- Preparation of the model compound.
- Devulcanization.
- Characterization.

**Figure 2:**
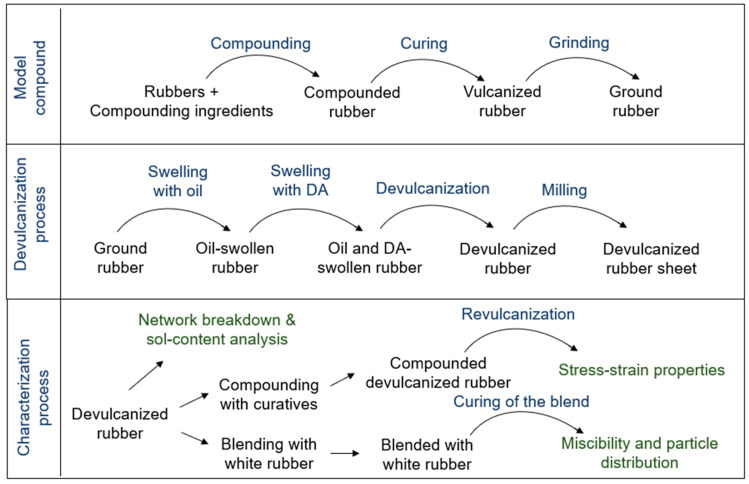
Flowchart of the experimental process.

A brief description of each component of the experimental process is described below and the flowchart is shown in Figure 2.

### 2.1. Model Compound Preparation

A SBR-BR-based silica-filled model tire tread compound was prepared as feed material for the devulcanization process. The kinetic viscosity of the TDAE oil at 40 °C was 331 mm^2^/s and at 100 °C it was 18.4 mm^2^/s. The different stages of the model compound preparation are described below.

#### 2.1.1. Materials

The model tire tread formulation is given in Table 1.

The list of the DAs for screening, reference DAs, and chemicals used for the characterization processes are described in Table 2.

#### 2.1.2. Compounding

Compounding was performed in two stages: the first step was performed in a 390 mL internal mixer (Model-350S) from Brabender GmbH & Co., Duisburg, Germany, followed by the final mixing on a lab scale two-roll mill (9 cm in diameter) of Schwabenthan GmbH & Co., Germany. The first step of mixing was conducted with a fill factor of 70%, initial temperature of 80 °C, and initial rotor speed of 70 rpm. During the initial step of the compounding process, the polymer underwent mastication to facilitate the incorporation of fillers and other compounding ingredients. As the temperature increased, the viscosity of the polymer decreased. Simultaneously, the addition of fillers led to an increase in viscosity. To ensure optimal reaction conditions for the silica–silane interaction, a temperature of 145 °C [30], known to be the optimum reaction temperature, was selected. Consequently, the starting temperature and rotor speed of the internal mixer were adjusted accordingly to achieve and maintain the desired temperature throughout the compounding process. Due to the high shear forces in the internal mixer during the addition of the polymer, filler, and other compounding ingredients, the temperature increased from 80 °C to 145 °C. Isothermal mixing was performed at 145 °C for 5 min by adjusting the rotor speed. The steps of the mixing process are detailed in Table 3.

The masterbatch, the material produced after the first mixing step, was kept for one day at room temperature before final mixing. Then, the compound was kept again for one day at room temperature before curing.

#### 2.1.3. Curing

The optimum cure time was measured in a RPA Elite from TA Instruments, New Castle, DE, USA. The samples were cured at 160 °C according to the T_95_ value. Compression molding was performed in an automatic press of Wickert Maschinenbau GmbH, Germany, with a 200 mm × 200 mm × 4 mm mold. After curing, the tensile strength of the vulcanized sheets was around 16 ± 1 MPa and the elongation at break was around 310 ± 30%.

#### 2.1.4. Chopping and Grinding

Chopping of the vulcanized sheets was performed with a bale cutter. The pre-treatment of the chopped samples was conducted by dipping them into liquid nitrogen for 4–5 min to achieve a temperature below the glass transition, followed by grinding at room temperature. Grinding was performed in a mechanical grinder from Fritsch, Germany, with a 0.7 mm mesh screen.

#### 2.1.5. Revulcanization

The devulcanized rubber samples were compounded and revulcanized for measuring the stress–strain properties. The revulcanization formulation is shown in Table 4.

The compounded devulcanized rubber was tested in the RPA 2000 Elite of TA Instruments, New Castle, DE, USA, at 160 °C for 30 min according to ASTM D7750-12 [31] to determine the optimum cure time. Sheets of 2 mm thickness were molded at 160 °C according to the T_95_ value in an automatic compression molding machine from Wickert Maschinenbau GmbH, Germany.

### 2.2. Devulcanization

The ground model tire tread rubber was the feed material for the devulcanization process. It was swollen with oil and DA before performing the devulcanization reaction. A brief description of the devulcanization process is given below.

#### 2.2.1. Swelling

The rubber granulate was first mixed with the process oil and after that with the DA at room temperature. After each addition, the samples were kept for one day at room temperature for swelling. Due to the high viscosity of the process oil, the rubber granulate was mixed with oil and manually stirred. The oil-swollen sample was then re-swollen with the DA and kept for one day for the migration of the DA into the particles.

#### 2.2.2. Devulcanization

The thermo-mechanical–chemical devulcanization process was performed in a Plastograph EC internal mixer with a volume of 50 cc from Brabender GmbH & Co., Germany. Non-intermeshing counter-rotating rotors and a telescopic ram were used. To minimize oxidation at high temperatures, the cavity was sealed with paraffin wax.

A two-roll mill from Schwabenthan, GmbH & Co., Germany, with a 200 mm length × 80 mm diameter was used at room temperature, with a 1.25 speed ratio at 30 rpm for the milling of the devulcanized rubber. The nip gap was reduced gradually from 1 to 0.1 mm, and the devulcanized rubber formed a band.

**Figure 3:**
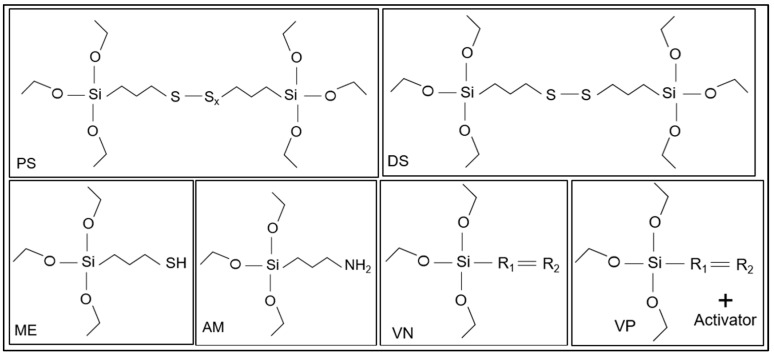
Chemical structure of the silanes used as DA (sample notations are explained in Table 2) (R1 and R2 = hydrocarbon chain).

According to pre-trial data, the variable and constant process conditions for the devulcanization were selected. For the reference sample, DPDS (diphenyl disulphide) and DBD (2-2′-dibenzamido-diphenyl disulphide) were used as DAs and processed according to the conditions obtained from the literature [32]. The sample details, including the variable and constant parameters for the DA screening trials, are shown in Table 5 and Figure 3.

#### 2.2.3. Characterization Process

The characterization process consisted of three parts: degree of devulcanization as deducted from the HorikxVerbruggen plot, stress–strain properties in tensile mode, and white rubber analysis.

#### 2.2.4. Degree of Network Breakdown

ASTM D 6814-02 [32] describes the standard procedure for the evaluation of the crosslink density according to the equilibrium volume swelling method and Flory–Rehner equation [33]. At first, the samples were extracted in acetone for the removal of the polar components, then dried. To remove the non-polar parts, the material was extracted with tetrahydrofuran.

The samples were then swollen in toluene at room temperature for 72 h, according to the ASTM standard. The volume fraction of the sample in the swollen gel (V_r_) was determined using the additivity rule of volumes as given in Equation (1) [33]:

(1) $$V_{r}=\frac{W_{r}/\rho_{r}}{W_{r}/\rho_{r}+W_{s}/\rho_{s}}$$

where W_r_ is the weight of the rubber specimen, W_s_ is the weight of the absorbed solvent, ρ_r_ is the density of the rubber, and ρ_s_ is the density of the solvent. The apparent crosslink density (V_c_) was calculated according to Flory–Rehner using Equation (2) [34]:

(2) $${\;V}_{c}=\frac{1}{2M_{c}}=\frac{-\left[\ln\left(1-V_{r}\right)+V_{r}+{\;\chi *\;V}_{r}^{2}\right]}{V_{s\;}\left(V_{r}^{\frac{1}{3}}-\frac{V_{r}}{2}\right)}$$

where V_r_ is the volume fraction of the polymer in the swollen specimen, χ is the Flory–Huggins polymer–solvent interaction parameter, M_c_ is the molecular weight between crosslinks, and V_s_ is the molar volume of the solvent. For the SBR–toluene system [35], the value of χ was 0.38 and the molar volume (V_s_) and the density values of toluene were 106.2 cm^3^/mol and 0.866 g/cm^3^, respectively. The percentage of network breakdown was calculated according to Equation (3) [36,37]:

(3) $$\;\mathrm{Network}\;\mathrm{breakdown}\;\mathrm{percentage}\;\left(\%\right)=\frac{V_{c1}-{\;V}_{c2}}{V_{c2}}$$

where V_c1_ and V_c2_ are the crosslink densities of the samples before and after devulcanization, respectively [33,36,37].

The crosslink density according to the Flory–Rehner equation was not the actual one in a filled compound. To determine the exact crosslink density, the Kraus correction was applied.

(4) $$\;V_{\mathrm{actual}}=\frac{V_{\mathrm{apparent}}}{1+k\times \;\Phi }$$

(5) $$\;\Phi =\frac{\mathrm{Weight}\;\mathrm{fraction}\;\mathrm{of}\;\mathrm{the}\;\mathrm{filler}\;\times \;\mathrm{density}\;\mathrm{of}\;\mathrm{the}\;\mathrm{compound}\;\times {\;W}_{b}}{\mathrm{Density}\;\mathrm{of}\;\mathrm{the}\;\mathrm{filler}\;\times {\;W}_{a}}$$

where V_apparent_ is the measured crosslink density according to the Flory–Rehner equation and V_actual_ is the actual crosslink density after correction for the filler. k is a constant for a given filler, Φ is the volume fraction of the filler in the specimen, W_b_ is the weight of the specimen before extraction, and W_a_ is the weight of the specimen after the extraction of all soluble material, such as polymer sol fraction, oil, and soluble chemical residues [36,37].

Considering the sol fraction of the feed material, the limiting lines of the random scission and crosslink scission were calculated according to the Horikx–Verbruggen method [37]. Plotting the values of the sol fraction and network breakdown of the devulcanizates in the graph, the type of network breakdown can be predicted. For one network breakdown versus a sol-content data plot, the average result of five samples was considered.

**Figure 4:**
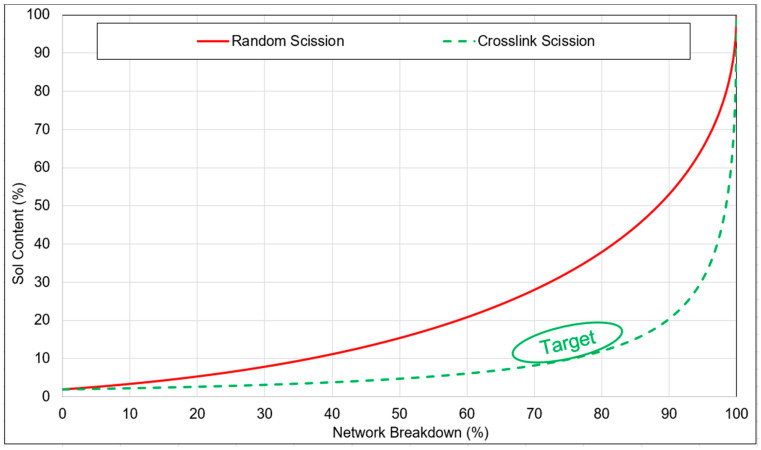
Sol content versus network breakdown percentage for main chain and crosslink scission according to the Horikx–Verbruggen plot.

In Figure 4, the marked green zone is the targeted area for the devulcanized rubber considering the minimum sol fraction with maximum devulcanization percentage. To achieve higher values, monosulphidic bonds have to be broken, which goes together with a higher degree of random scission. Moreover, bound rubber cannot be solved, limiting the sol content. FTIR was performed with a Spectrum 100 spectrometer from PerkinElmer, Waltham, MA, USA.

#### 2.2.5. Stress–Strain Properties

Stress–strain properties of the revulcanized rubber were measured with a Z010 tensile tester manufactured by Zwick Roell GmbH & Co., Germany, according to ASTM D412 [38]. For each sample, a total of seven tensile dumbbells were tested and, among those, five significant values were plotted in one data point with their average values and error bars.

#### 2.2.6. Miscibility

After the devulcanization process, there may still be undevulcanized particle cores in the devulcanizate. Devulcanized polymer chains are homogeneously miscible with a compatible polymer or compound; however, undevulcanized particle cores are not under the same conditions. Considering this, it is important to analyze the number, size, and total area of these undevulcanized particle cores. For this purpose, white rubber analysis (WRA) was developed. In this quantitative analytical method, the devulcanizate was blended with a bright-white polybutadiene-based compound colored with titanium dioxide. The white colorant was judiciously selected to create a high contrast between the background and devulcanizate, which led to a quantitative characterization process.

White rubber compounds are polybutadiene and titanium dioxide based. The samples were prepared by mixing 10% devulcanizate into this white rubber compound. This resulted in a gray compound, in which the remaining non-devulcanized particles were visible as brownish spots. The digital analysis of the particles and particle size distribution was performed using a VHX 5000 digital microscope from Keyence.

#### 2.2.7. Thermogravimetric Analysis

Thermogravimetric Analysis (TGA) was performed using a TGA 550 instrument, supplied by TA Instruments, New Castle, DE, USA. The test was conducted in a nitrogen atmosphere with a flow rate of 50 mL/min, ramp speed of 10 °C/min, and ranging from 30 °C to 600 °C. Subsequently, the test was continued in an air atmosphere comprising 70% N_2_ and 30% O_2_, with the same flow rate and ramp speed, from 600 °C to 900 °C. Equilibration was performed at three different temperatures, namely, 30 °C, 600 °C, and 900 °C, each for a duration of five minutes.

## 3. Results and Discussion

This part was classified into the three main properties: degree of network breakdown, stress–strain properties, and homogeneity. Finally, the reaction mechanism was elucidated.

### 3.1. Network Breakdown

**Figure 5:**
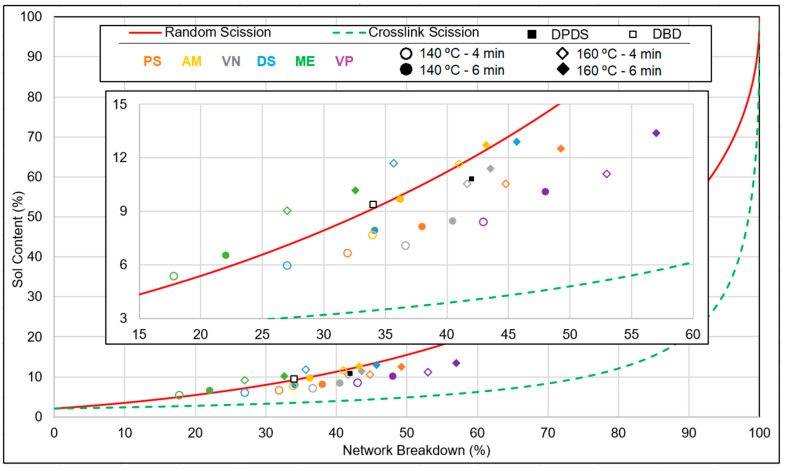
Horikx–Verbruggen plot: DA screening (sample notations are explained in Table 2).

In Figure 5, the network breakdown versus sol-content data of six silane devulcanized samples along with DPDS and DBD devulcanizates as reference is plotted. Network breakdown and sol content gradually increased with the increasing residence time and temperature. It can be concluded that, on an average, VP showed the highest efficiency and ME was the least efficient one in terms of network breakdown and sol content. Using VP as DA, the network breakdown reached 57.1% at a sol content of 13.4%: this was much lower than the random scission limit. The more efficient crosslink scission compared to the other chemicals tested as DAs must have been due to the presence of the alkenyl moiety and activator in VP. VN resulted in a network breakdown of app. 45% on average with a sol content of 2% for the best processing conditions.

With PS, the network breakdown percentage increased from 25% to 40% with an increasing residence time of 4 to 6 min, for a devulcanization temperature of 140 °C. When the temperature was increased to 160 °C at a residence time of 6 min, the sol content increased from 6% to 10% and the network breakdown percentage increased to 53%. The breaking of the crosslinks and reaction with the DA to hamper the recombination and to form sulphur–silane bonds was a probable reason for the higher degree of devulcanization. For the samples devulcanized with DS, AM, and ME, the data points were mostly close and above the random scission line. The points are on the left side of the graph, which indicates insufficient devulcanization of the granulate. It can be concluded that the di-sulphidic, mercapto, and amino moieties are less efficient than the alkenyl and polysulphide groups in this process window.

### 3.2. Stress–Strain Properties

**Figure 6:**
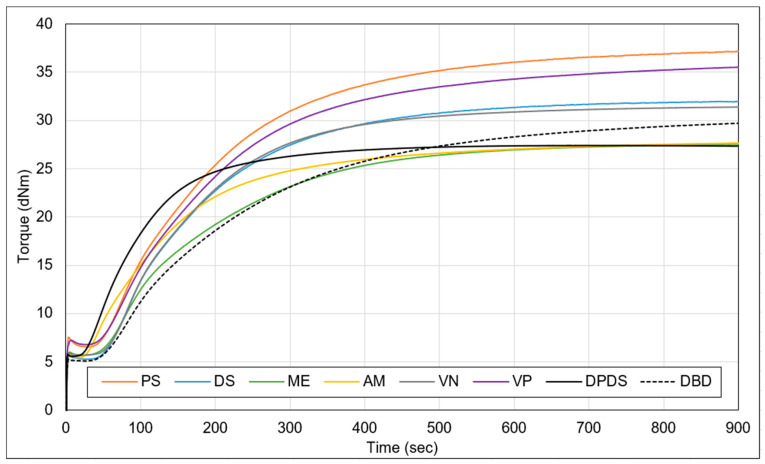
Cure curves of the compounded devulcanizates (sample notations are explained in Table 2).

Based on Figure 6, the results reveal that the increase in curing torque is lowest for the DPDS samples, highest for the PS samples, and followed by the VP samples. This can be attributed to the presence of unreacted polysulphide moieties in the PS samples, which contributed to the formation of additional crosslinks and resulted in a greater curing torque increase. Furthermore, compared to the VN samples, the VP samples exhibited increased curing torque due to the contribution of the activator. Additionally, the samples with a lower degree of network breakdown exhibited a lower increase in torque, indicating less formation of additional crosslinks.

**Figure 7:**
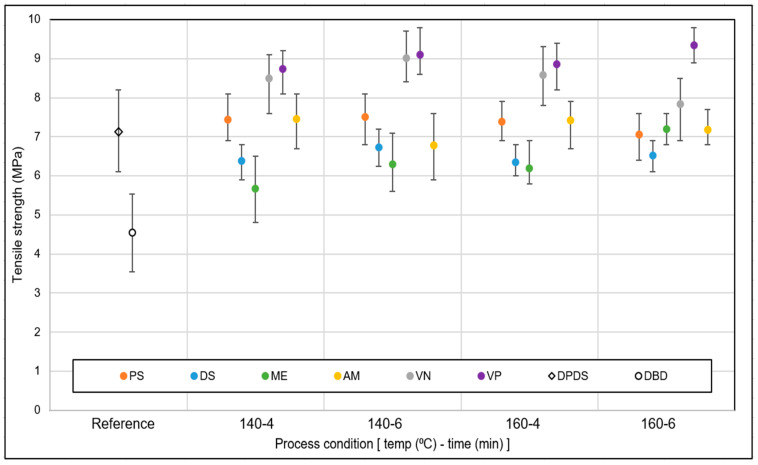
Tensile strength: DA screening (sample notations are explained in Table 2).

The comparative analysis of the tensile strength of the revulcanized samples is shown in Figure 7. The tensile strength of the revulcanizate varied with increasing time and temperature. The best stress–strain properties were obtained for the VN and VP samples. The tensile strength was in the range of 8 to 9.4 MPa. From these screening trials, it can be concluded that VP and VN as DAs resulted in the best mechanical strength.

**Figure 8:**
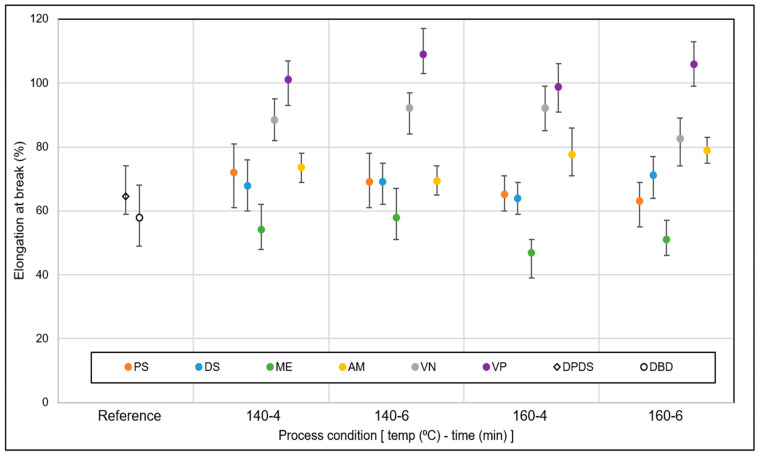
Elongation at break: DA screening (sample notations are explained in Table 2).

A comparative analysis of the elongation at break values of the revulcanized samples is shown in Figure 8. For the reference DAs as well as for PS, DS, and AM devulcanized samples, elongation at break was in the range of 60% to 80% and did not show any significant change with increasing time and temperature. For ME as DA, the range was between 50% to 65%, and for VN the range was 75% to 95%. The best elongation at break values were found for the VP samples, which were in the range of 95% to 110%.

From this screening, it can be concluded that VP resulted in the best stress–strain properties, followed by VN. Considering the tensile strength of the feed material, the tensile strength recovery was around 50–55%. This fits well with the findings of the network breakdown analysis: VP showed the best performance as DA.

### 3.3. Miscibility Analysis

**Figure 9:**
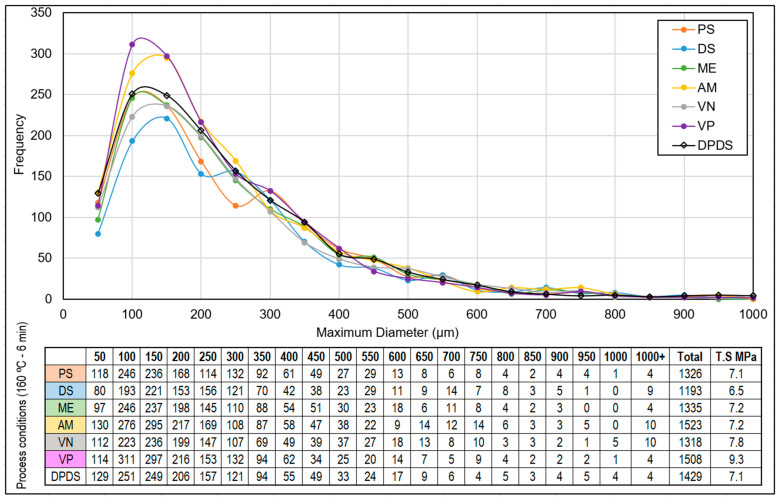
White rubber analysis: DA screening (sample notations are explained in Table 2).

The homogeneity of the DA optimization trials was elaborated in the white rubber analysis. From the statistical analysis of the brownish devulcanizate dispersed in the white rubber matrix, the total number of visible particles and the particle size distribution were analyzed. The series of samples devulcanized at 160 °C for 6 min was selected. The comparative results for devulcanizates using different DAs are shown in Figure 9. 

For the samples devulcanized with VP or AM, the particle size distribution was on the finer side, and the total number of visible particles was higher than for all other samples. The samples devulcanized with DS, PS, VN, ME, and DPDS were comparatively coarser with a lower number of visible particles. This shift in the particle size distribution was an effect of the devulcanization efficiency and mechanical grinding processes.

**Figure 10:**
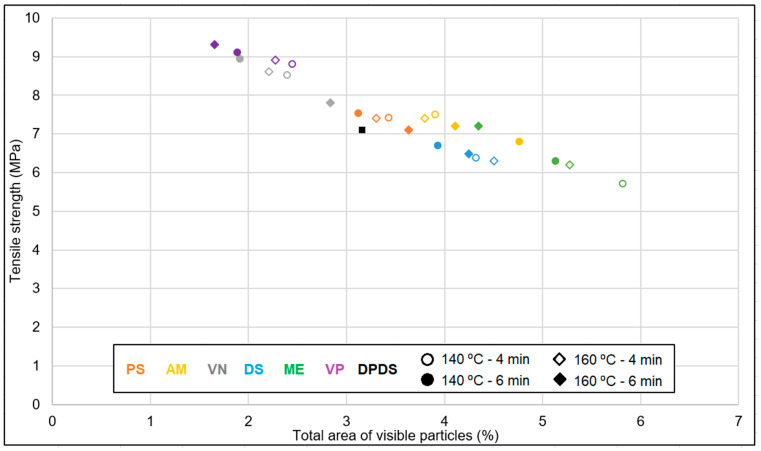
Correlation between tensile strength and total area of visible particles (sample notations are explained in Table 2).

In Figure 10, the total area of visible particles (TAVPs) is plotted against the tensile strength (TS). With an increasing devulcanization efficiency, as indicated by a smaller TAVP, the TS improved. For ME devulcanized samples, the TAVP had the highest value, resulting in the lowest TS, and for VP as devulcanization aid, the TAVP was the minimum, resulting in the best TS, fitting with the explanation mentioned above.

### 3.4. Thermogravimetric Analysis

**Figure 11:**
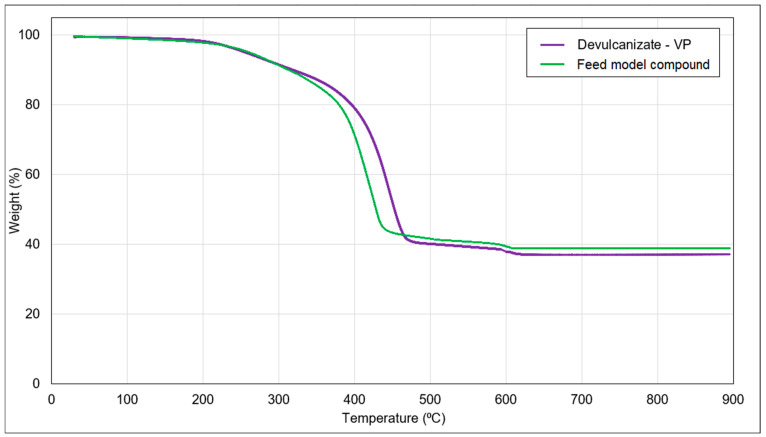
TGA analysis of the feed model compound and VP devulcanizate (sample notation is explained in Table 2).

Figure 11 illustrates the TGA analysis curve of the VP devulcanized rubber sample, which comprises approximately 45–50% of the polymer. The volatile content of the sample was approximately 10% and no significant weight loss was observed up to 200 °C. This indicated that the devulcanizate exhibited thermal stability within typical application temperatures. The residual content observed in the TGA curve represented the contribution from silica, which accounted for approximately 35%, while the concentration of zinc oxide was around 2–3%. In comparison to the TGA of the feedstock, the devulcanizate had higher content of volatiles due to the addition of oil and low-molecular-weight polymer fragments. It had a lower residue as the addition of oil resulted in a lower content of inorganic components.

### 3.5. Model Devulcanization Mechanism

**Figure 12:**
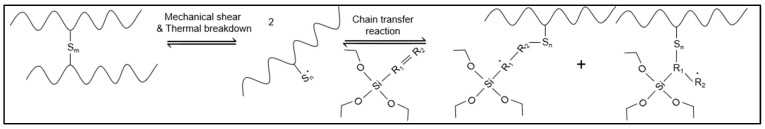
Chain-transfer reaction between alkenyl silane and polymer crosslinks.

The alkenyl silane with the activator presented the best performance within this group of potential DAs. From a mechanistic view, one of the reasons was the presence of the unsaturated carbon–carbon bond. The proposed reaction mechanism is that the alkenyl silane and polymer react and experience a chain-transfer reaction, in which the alkenyl moiety becomes attached to the broken crosslinks and restricts a further recombination, as shown in Figure 12.

**Figure 13:**
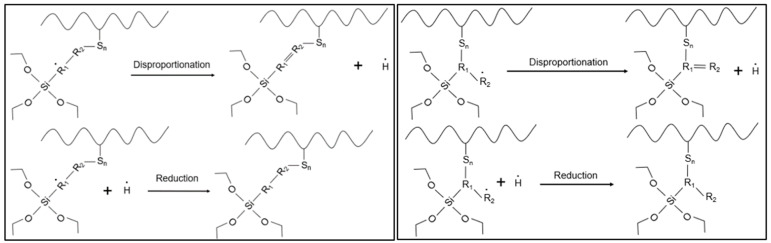
Disproportionation reaction between alkenyl silane and polymer crosslinks.

After the chain transfer, the molecule experiences a disproportion reaction to achieve greater chemical stability. The activator in the alkenyl silane improved the reactivity of the latter during devulcanization, as shown in Figure 13. An additional mechanistic investigation is necessary to prove this reaction hypothesis for the devulcanization.

**Figure 14:**
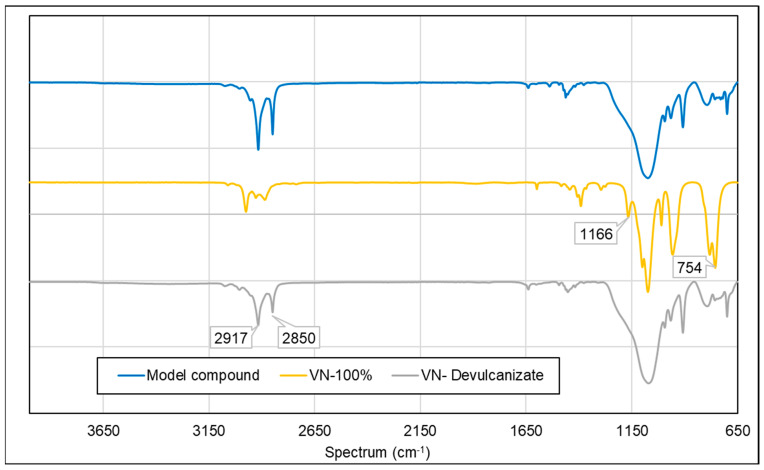
FTIR spectroscopy of the model compound, VN, and VN devulcanizate (sample notations are explained in Table 2).

The model compound used as the feed material for the devulcanization process contained carbon–carbon double bonds: the base polymers were unsaturated rubbers, such as SBR and BR. In the preparation of the model compound, Si 266^®^ was employed as a coupling agent to enhance silica reinforcement containing ethoxy groups. VN contains both ethoxy groups and carbon–carbon double bonds. The comparative peak intensities can provide an insight into the progression of the devulcanization reaction, as shown in Figure 14.

In the devulcanizate, a reduction in the intensity was observed for the peaks at 2850 cm^−1^ and 2917 cm^−1^, which corresponded to the C–H bending of the alkanes and alkenes. The devulcanization reaction did not typically result in the formation of new moieties in the final product; it was primarily a chain-transfer reaction. No other significant peaks were observed [39].

In comparison to pure VN, several peaks disappeared in the devulcanized sample. A peak at 1166 cm^−1^, corresponding to C–O stretching, was noted, indicating the involvement of this functional group in the devulcanization process. Additionally, a peak at 754 cm^−1^, associated with the C=C bending of alkene, was detected, signifying the reactivity of VN during devulcanization. These observations provide evidence of the reactivity and transformation of VN during the devulcanization process [39].

## 4. Conclusions

The devulcanization efficiencies of the different silanes in terms of mechanical properties, network breakdown, and miscibility were comparatively analyzed in this study. The SBR-silica network is the main challenge for passenger car tire rubber devulcanization, and a potential solution was developed by using alkenyl silane as a devulcanization aid (DA). The efficiency of this silane as DA was further improved by the presence of an activator; this combination turned out to be the most promising DA for passenger car tire rubber devulcanization.

Alkenyl silane and alkenyl silane with activator devulcanized samples showed the best mechanical and network breakdown properties, followed by the ones devulcanized with polysulphidic and amino silane. Compared to other silanes, the alkenyl and the variant with the activator showed a higher crosslink-to-random-scission ratio, resulting in a better devulcanizate quality. A greater extent of devulcanization in rubber leads to an increased number of free polymer chains, resulting in enhanced miscibility. Conversely, reduced devulcanization leads to a reduced presence of free polymer chains, resulting in decreased miscibility and an increased quantity of immiscible particles. A linear correlation was established between the devulcanizate quality in terms of tensile strength and the total area of visible, insufficiently devulcanized particles for all silane devulcanized samples.

Considering the properties of the feed material, the recovery of the tensile strength was around 50–55%, and the network breakdown percentage was 55–60%: these are rather high percentages for an SBR–silica system due to the strong filler–polymer network. An optimization of the process parameters is required for the further improvement of the devulcanizate quality. A reaction mechanism was proposed; however, further research on and the analysis of the devulcanization process and reactions are required.

## Figures and Tables

**Table 1:** Compounding formulation of the model tire tread compound.

Function	Ingredient	Trade Name	Supplier	Quantity (phr)
Polymer	SSBR ^1^	Sprintan 4601	Trinseo	70
	BR ^2^	CB 24	Arlanxeo	30
Filler system	Silica	ULTRASIL^®^ 7000 GR	Evonik	80
	Silane	Si 266^®^	Evonik	5.8
Activators	Zinc oxide	Merck Zinc Oxide	Sigma-Aldrich	3
	Stearic acid	Merck Stearic Acid	Sigma-Aldrich	2
Plasticizer	TDAE oil ^3^	Vivatec	H & R	25
Curing system	Curing aid	Merck Sulphur	Sigma-Aldrich	1.5
	Pri. accelerator	Santocure CBS	Flexsys	1.7
	Sec. accelerator	Perkacit DPG	Flexsys	2.5

^1^ Solution-polymerized styrene butadiene rubber; ^2^ polybutadiene rubber; ^3^ treated distillated aromatic extracted oil.

**Table 2:** Chemicals used for devulcanization and characterization processes.

Ingredient Notation	Chemical Identification	Supplier
PS	Polysulphide: bis-triethoxy-silyl-propyl tetrasulphide	Evonik
DS	Bis-triethoxy-silyl-propyl disulphide	Evonik
ME	Triethoxy-silyl-propyl mercapto silane	Evonik
AM	Triethoxy-silyl-propyl amino silane	Evonik
VN	Triethoxy-silyl-alkenyl silane	Evonik
VP	Triethoxy-silyl-alkenyl silane with activator	Evonik
DPDS	Di-phenyl di-sulphide	Sigma-Aldrich
DBD	Di-benzamido di-phenyl di-sulphide	Sigma-Aldrich
Acetone	Acetone	Boom Lab.
Toluene	Toluene	Boom Lab.
THF	Tetrahydrofuran	VWR Chem.
TiO_2_	Titanium dioxide	Sigma-Aldrich
MBTS	Mercapto-benzothiazole sulphenamide	Sigma-Aldrich

**Table 3:** Mixing process of the model tire tread compound.

Masterbatch (First) Step		Final (Second) Step	
Action	Time [mm:ss]	Action	Time [mm:ss]
Polymer	00:00–00:30	Masterbatch	-
Mastication	00:30–01:30		
½ Silica + silane	01:30–02:00	Mixing	00:00–02:00
Mixing	02:00–03:00		
½ Silica + silane	03:00–03:30	Curatives	02:00–02:30
+other additives			
Mixing (140–150 °C)	03:30–04:30		
Ram sweep	04:30–05:00	Mixing	02:30–09:00
Mixing (target 145 °C)	05:00–09:00		
Discharge and sheeting	-	Discharge	-

**Table 4:** Revulcanization formulation.

Function	Component	Weight (%)
Base polymer	Devulcanized rubber sample	100
Activators	Zinc oxide	4
	Stearic acid	2
Vulcanization agent	Sulphur	2
Accelerator	CBS	1

**Table 5:** Details of devulcanization process optimization.

**Devulcanization Aid Screening**								
**No.**		**Name of DA**	**Temperature (°C)**		**Time (min)**	**Constant Parameters**		
1–4		PS	140 °C 160 °C		4 min 6 min	DA (silane): 5% (*w*/*w*) Process oil: 5% (*w*/*w*) Shear rate: 150 RPM Fill factor: 80%		
5–8		DS						
9–12		ME						
13–16		AM						
17–20		VN						
21–24		VP						
**Benchmark Samples**								
**No.**	**DA**	**DA Conc.**	**Swelling**	**Oil**	**Temperature**	**Speed**	**Fill Factor**	**Time**
1	DPDS	30 mmol	1 day at 65 °C	5% TDAE	220 °C	50 rpm	60%	6 min
2	DBD							

## Data Availability

Not applicable.
